# Supplementary material for: A neurotrophin functioning with a Toll regulates structural plasticity in a dopaminergic circuit
Source: eLife. 2024 Dec 20;13:RP102222. doi: 10.7554/eLife.102222 (PMC11661795; doi:10.7554/eLife.102222)
Supplement: Figure 5—figure supplement 1—source data 2. [file elife-102222-fig5-figsupp1-data2.zip › Figure 5 supplement 1 - source data 2 pdf- eLife-RP-RA-2024-102222 VOR.pdf]

Figure 5 figure supplement 1 - source data 2

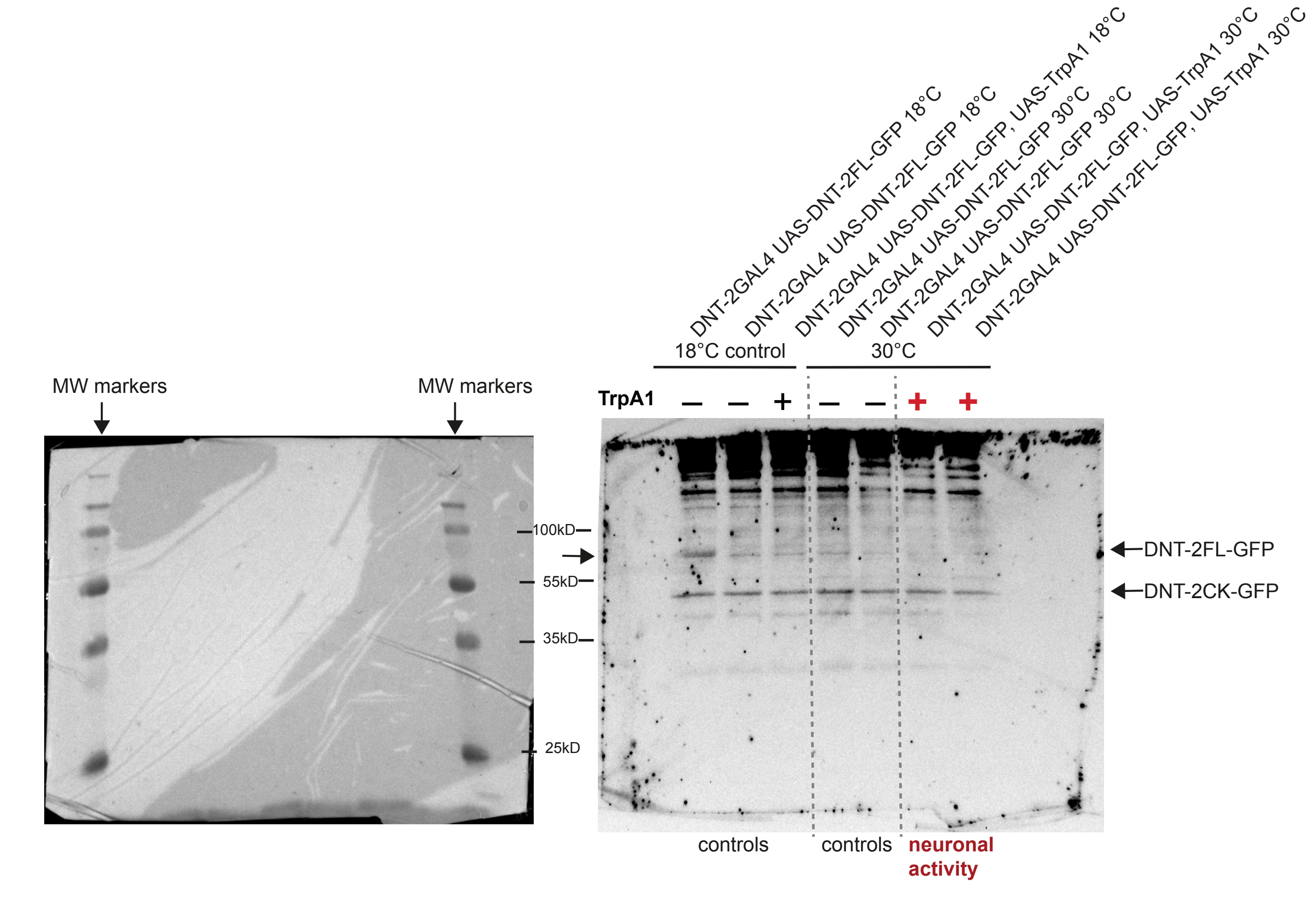

Original membranes corresponding to Figure 5 figure supplement 1B. Left: molecular weight (MW) markers. Right: uncropped membrane, with molecular weight markers indicated (kilo Dalton) and arrows indicate the positions of DNT-2-FL-GFP and DNT-2-CK-GFP. Genotypes indicated on top of lanes.
