# Supplementary figures and images for: A neurotrophin functioning with a Toll regulates structural plasticity in a dopaminergic circuit
Source: eLife. 2024 Dec 20;13:RP102222. doi: 10.7554/eLife.102222 (PMC11661795; doi:10.7554/eLife.102222)

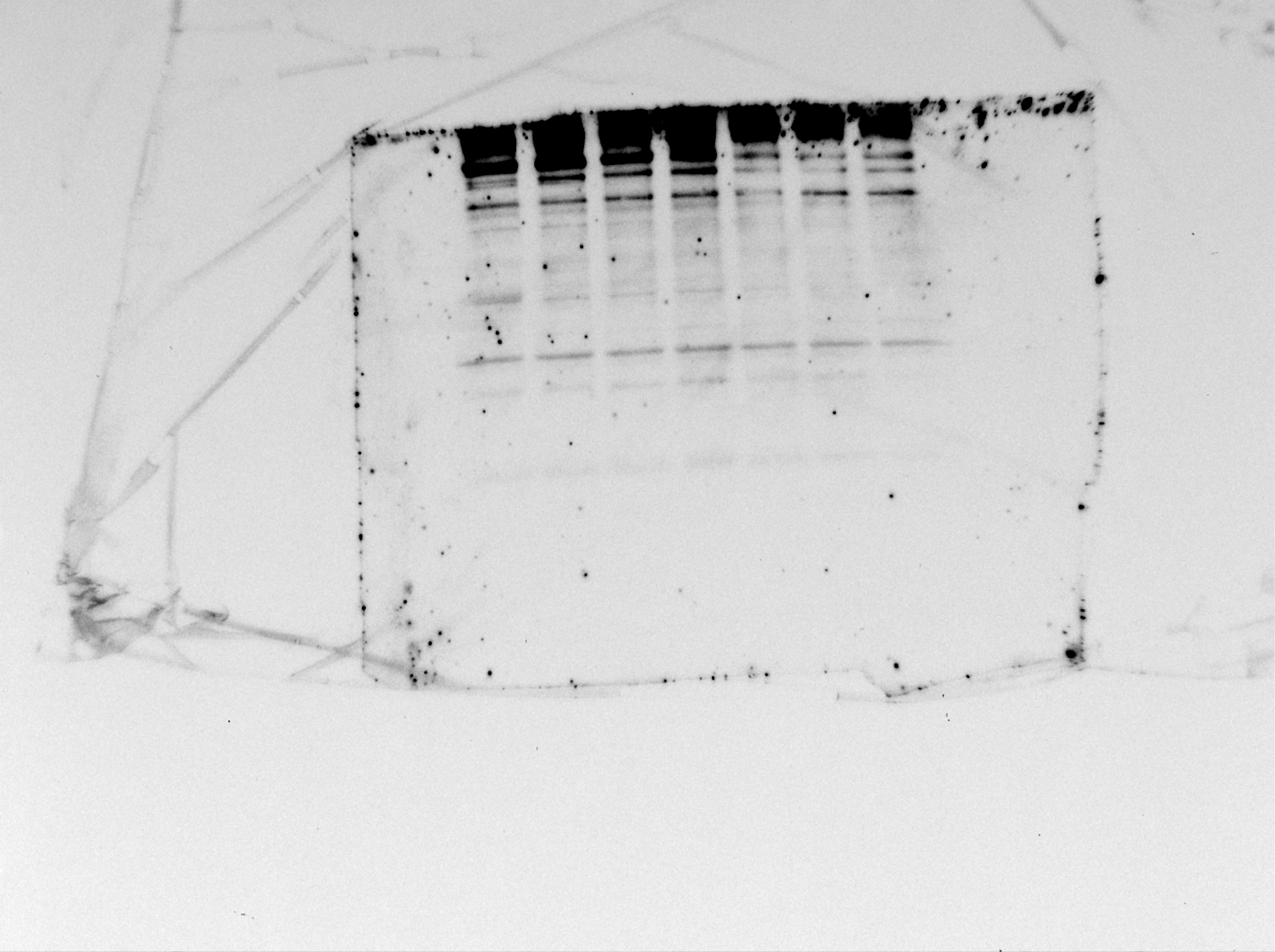

Supplement: Figure 5—figure supplement 1—source data 3. [file elife-102222-fig5-figsupp1-data3.zip › Figure 5 figure supplement 1 - source data 2 original file eLife-RP-RA-2024-102222 VOR/DNT2Gal4 UASDNT2FLGFP withdTrpA1 2 eLife-RP-RA-2024-102222 VOR.tif]
